# Machine Learning for Identification of Cirrhosis in Autoimmune Hepatitis Using Routine Biomarkers and Liver Elastography: Development and External Validation of an Interpretable Classification Model

**DOI:** 10.3390/biomedicines14092045

**Published:** 2026-09-11

**Authors:** Nazugum Ashimova, Symbat Abzaliyeva, Araylym Maldanova, Madina Suleimenova, Andreas Teufel, Alexander Nersesov

**Affiliations:** 1 Department of Gastroenterology, Asfendiyarov Kazakh National Medical University, Almaty 050012, Kazakhstan; nazugumashimova90@gmail.com (N.A.); alexander.nersesov@gmail.com (A.N.); 2 Institute of Gastroenterology, Hepatology and Metabolism “Interna Clinic”, Almaty 050000, Kazakhstan; maldanova97@mail.ru; 3 Department of Big Data and Artificial Intelligence, Faculty of Information Technology, Al-Farabi Kazakh National University, Almaty 050040, Kazakhstan; 4 Department of Information Systems, International Information Technologies University, Almaty 050040, Kazakhstan; 5 Department of Internal Medicine A, University of Greifswald, 17489 Greifswald, Germany; andreas.teufel@med.uni-greifswald.de

**Keywords:** autoimmune hepatitis, liver cirrhosis, machine learning, biomarkers, classification, SHAP, artificial intelligence in medicine

## Abstract

**Background:** Autoimmune hepatitis (AIH) is a chronic immune-mediated liver disease that may progress to cirrhosis. This study aimed to develop and externally validate interpretable machine-learning models for the classification of prevalent cirrhosis in patients with AIH. **Methods:** The development cohort included 55 patients with biopsy-confirmed AIH. Cirrhosis was defined histologically as F4, whereas F0–F3 was classified as non-cirrhosis. Logistic Regression with L2 regularization, Random Forest, and XGBoost were evaluated. The original stratified 70/30 hold-out analysis was retained, and repeated stratified five-fold cross-validation with 20 repeats was additionally performed to assess internal stability. Primary external validation was performed in an independent histology-matched cohort of 42 patients. Models were applied without refitting, recalibration, or threshold optimization. Discrimination, probabilistic accuracy, calibration, and threshold-dependent classification metrics were evaluated. **Results:** In the original held-out test set, AUROC was 0.900 for Logistic Regression with L2 regularization, 0.830 for Random Forest, and 0.890 for XGBoost. In repeated cross-validation, mean AUROC was 0.878 ± 0.021, 0.914 ± 0.015, and 0.896 ± 0.015, respectively. In the primary external validation cohort, Random Forest showed the highest numerical discrimination (AUROC 0.810; 95% CI, 0.653–0.933), followed by XGBoost (0.728; 95% CI, 0.566–0.878) and Logistic Regression with L2 regularization (0.716; 95% CI, 0.545–0.875). Random Forest also had the lowest Brier score (0.188). However, confidence intervals were wide and overlapping. Elastography stage alone achieved an AUROC of 0.745, and the numerical improvement of the full Random Forest model was not statistically clear. **Conclusions:** Machine-learning models integrating routinely available clinical, biochemical, immunological, and elastography-related variables showed preliminary external transportability for the classification of prevalent cirrhosis in AIH. However, the small development and validation cohorts, uncertainty in calibration, and lack of a clearly demonstrated incremental advantage over elastography alone indicate that larger prospective multicenter studies are required before clinical implementation.

## 1. Introduction

Autoimmune hepatitis (AIH) is a chronic immune-mediated liver disease characterized by persistent hepatocellular inflammation, interface hepatitis, and progressive fibrosis that may culminate in cirrhosis and liver failure [1]. Early identification of patients at increased risk of cirrhosis is essential for timely treatment optimization and prevention of irreversible liver damage. However, the disease course in AIH is heterogeneous, and progression is shaped by complex interactions among biochemical, immunological, and clinical factors [2].

Histological fibrosis staging systems, MELD-based scores, and individual immunoserological markers provide important diagnostic and prognostic information, but they only partially reflect the multidimensional nature of AIH. Noninvasive methods, such as transient elastography and serum fibrosis indices (e.g., APRI, FIB-4), have improved the assessment of fibrosis in chronic liver diseases, including AIH; however, their diagnostic performance remains variable, and they may be insufficient for the accurate detection of early cirrhosis. Autoantibodies remain an important component of the diagnosis of autoimmune hepatitis. ANA and ASMA are most commonly detected in type 1 AIH, whereas antibodies to LKM-1 and LC-1 are characteristic of type 2 AIH [3]. Several studies have shown that transient elastography is an effective, noninvasive method for assessing fibrosis in autoimmune hepatitis and, in general, outperforms serum markers of fibrosis. However, elastography cannot completely replace a liver biopsy, especially when it is necessary to assess the histological activity of the disease or confirm remission [4,5,6]. It is important to note that cirrhosis may be overestimated when using macroscopic and imaging methods and underestimated in patients with normal transaminase and IgG levels, as demonstrated by in a previous study [7]. As a result, reliably identifying patients at the highest risk of disease progression using only traditional approaches remains a challenging task [8,9].

In recent years, machine learning (ML) has emerged as a promising tool in hepatology for integrating diverse clinical, biochemical, and imaging data and uncovering patterns associated with disease progression [10]. ML-based models have shown strong performance in predicting advanced fibrosis, hepatocellular carcinoma, and short-term mortality in various chronic liver diseases, often outperforming conventional statistical scores [11]. Recent studies have also explored AI-based feature fusion and ensemble-learning approaches for liver disorder prediction, further supporting the potential of integrated machine-learning strategies for heterogeneous hepatology data [12].

In our previous study [13], machine-learning models based on routine clinical and laboratory variables were developed for the detection of advanced fibrosis (F3–F4 vs. F0–F2) in patients with AIH; however, the models were limited to internal validation.

Despite the growing application of ML in hepatology, independently validated models specifically addressing cirrhosis in AIH remain limited. AIH-specific immunological markers, including ANA, ASMA, IgG, and γ-globulins, may provide additional information alongside routine clinical and biochemical variables [14,15]. Previous work has also demonstrated the importance of external validation and model interpretability when ML models are developed in relatively small cohorts [16]. This is particularly relevant to AIH, where well-characterized cohorts are often limited and independent validation is essential for assessing model generalizability.

Thus, this study aimed to develop and externally validate interpretable machine learning models for classification of cirrhosis in patients with AIH, using routinely available clinical, biochemical, immunological, and elastography markers. By combining machine learning algorithms, external validation, and model interpretability analyses, we sought to assess whether these markers can support accurate classification of cirrhosis status in patients with AIH.

## 2. Materials and Methods

This study was carried out at the Institute of Gastroenterology, Hepatology, and Metabolism in Almaty, Republic of Kazakhstan, within the framework of the intramural research grant of Asfendiyarov Kazakh National Medical University (Order No. 483, 18 July 2024). The study was conducted in accordance with the Declaration of Helsinki, and approved by the Institutional Review Board of Asfendiyarov Kazakh National Medical University (protocol code 163); date of approval: 29 April 2025.

Adult patients with a probable or confirmed diagnosis of AIH according to the simplified IAIHG criteria and a liver biopsy were consecutively enrolled between May 2025 and March 2026. Inclusion criteria included age ≥ 18 years and a confirmed diagnosis of AIH based on serological and histological data. Only patients with complete clinical, laboratory, histological, and transient liver elastography data were included in the final analysis.

We excluded individuals with concomitant hepatitis B or C virus infection, significant alcohol consumption, other chronic liver diseases such as MASLD, primary biliary cholangitis, or primary sclerosing cholangitis, Wilson disease, hemochromatosis, and hepatocellular carcinoma. The development cohort included 55 patients with biopsy-confirmed autoimmune hepatitis. This cohort represents a biopsy-characterized subgroup of the 240-patient cohort previously reported by our group [13] with overlap in patients and several predictors including age, disease duration, platelet count, ALT, AST, bilirubin, albumin, IgG, and histological parameters. Unlike the previous study, which evaluated advanced fibrosis (F3–F4 vs. F0–F2), the present study specifically focused on cirrhosis (F4 vs. non-cirrhosis), using histology as the reference standard. The predictive models were trained exclusively on this development cohort and then applied to an external validation cohort without retraining, recalibration, feature reselection, or threshold optimization. An independent external validation cohort comprised patients evaluated between 2019 and 2025 at the Hepatology Center in Almaty, Kazakhstan. The external database contained 171 AIH patient records. Before primary external validation, record-level screening was performed to ensure independence from the development cohort, and overlapping records were excluded. This resulted in 116 non-overlapping external records. For the primary external validation analysis, an available histological fibrosis stage was required to ensure consistency of the reference standard with the development cohort. This yielded 42 independent patients, including 16 patients with F4 cirrhosis and 26 patients with F0–F3 fibrosis.

For the primary modelling analysis, prevalent histological cirrhosis was defined as F4, whereas F0–F3 was classified as non-cirrhosis. Histological fibrosis stage was used to define the outcome and was not supplied to the machine-learning models as a predictor.

Liver biopsy was performed percutaneously under ultrasound guidance using an 18-gauge needle. Samples with a core length of at least 1.5 cm and eight or more portal tracts were considered adequate. Fibrosis was assessed using the METAVIR system (F0–F4) and the modified Ishak score, where 0 points indicated the absence of fibrosis and 6 points indicated cirrhosis. Necroinflammatory activity was assessed according to standard criteria. Histological features typical of autoimmune hepatitis, including interface hepatitis and lymphoplasmacytic infiltrates, were assessed by experienced pathologists blinded to the elastography and machine learning results. For modeling purposes, cirrhosis was defined as METAVIR stage F4 and/or Ishak stage 5–6 and was used as a binary outcome (cirrhosis vs. no cirrhosis).

Transient elastography was performed by an experienced operator using the FibroScan 630 Expert device (Echosens, Paris, France). The examinations were conducted in accordance with the manufacturer’s recommendations: at least ten valid measurements, a success rate of 60% or higher, and an interquartile range-to-median ratio of 30% or less.

At the time of the biopsy, the treatment status of patients in the cohort was heterogeneous. Three patients (5.5%) were receiving prednisolone based on clinical indications (for less than one month). The remaining 52 patients (94.5%) were taking ursodeoxycholic acid until the diagnosis was confirmed. The use of UDCA prior to diagnostic verification reflected empirical therapy during the diagnostic evaluation phase in patients with unexplained cytolysis and was not associated with the presence of overlap syndromes (AIH-PBC; AIH-PSC). The diagnosis of AIH was confirmed by clinical, immunological, and histological criteria, which allowed for the formation of a diagnostically homogeneous study cohort.

Before analysis, all data were anonymized (de-identified). Continuous variables were checked for implausible values and outliers; extreme values falling outside physiologically plausible ranges were cross-referenced with the source data and, when necessary, treated as missing. To examine the relationships between fibrosis and individual biomarkers, we calculated Spearman’s rank correlation coefficients for liver stiffness, biochemical markers, immunological parameters, and histological stage of fibrosis, and visualized the resulting matrix as a heat map. A two-sided *p* value < 0.05 was considered statistically significant.

To classify cirrhosis, we developed supervised machine learning models using clinical, laboratory and elastography data available in routine practice. The binary target variable represented the presence or absence of cirrhosis at the time of assessment, as determined by the predefined histological reference standard. The dataset was randomly divided into a training set (70%) and a test set (30%) using stratified sampling to maintain a roughly equal proportion of patients with and without cirrhosis in both subgroups. Three classifiers were implemented: logistic regression with L2 regularization, random forest, and XGBoost. Model development was performed in Python 3.13.5 and R version 4.3.1. For the Random Forest and XGBoost algorithms, we tuned key hyperparameters such as the number and depth of trees, learning rate, and the minimum number of samples per leaf using stratified cross-validation on the training dataset, optimizing the area under the receiver operating characteristic curve (AUC). Sensitivity, specificity, positive predictive value (PPV), and negative predictive value (NPV) were calculated from the confusion matrix on the holdout test dataset using the default classification threshold (0.5). The 95% confidence intervals (95% CI) for these metrics were calculated using Wilson’s estimation method. For internal evaluation, models were trained on the training subset and evaluated on the holdout test subset. For external validation, the final models were retrained on the full development cohort and then applied to an independent external validation cohort.

Based on data from the training set, logistic regression models with L2 regularization, random forest, and XGBoost were developed. For internal evaluation, the training set was randomly split into training and test subsets using stratified sampling. For external validation, the final models were trained on the entire training dataset and then applied to an independent external validation dataset.

The final harmonized predictor set comprised 14 variables: age group, sex, disease duration, ANA, ASMA, IgG, γ-globulins, pre-treatment elastography stage, ALT, AST, total bilirubin, albumin, INR, and platelet count. Histological activity was excluded because it was derived from the same biopsy used to establish the reference outcome. Ethnicity, harmful habits, histological fibrosis stage, Child–Pugh class, MELD-Na, and cirrhosis-related complications were not included in the machine-learning predictor set.

The model’s performance was evaluated using accuracy, the F1 score, and AUC on the test dataset. Receiver operating characteristic (ROC) curves were plotted to compare the discriminatory power of the three algorithms. Feature importance was determined using tree-based models to assess the relative contribution of each biomarker to the classification of cirrhosis status. Predefined codes denoting unavailable or unmeasured values were treated as missing. Missing predictor values were imputed using values estimated exclusively from the corresponding training data. For Logistic Regression with L2 regularization, standardization was fitted only on the training data and then applied unchanged to validation observations. Random Forest and XGBoost were fitted without standardization. All preprocessing steps were implemented within the modelling pipeline so that observations used for evaluation did not contribute to preprocessing parameters. Feature importance and SHAP analyses were performed for the final Random Forest model to characterize the relative contribution of the harmonized predictors to model output. Key parameters used in our study are presented in Table 1.

## 3. Results

Table 2 presents the baseline characteristics of the development cohort and the full external clinical cohort. The cohorts showed similar distributions for several demographic and laboratory characteristics, whereas differences were observed in γ-globulin status, albumin levels, and the prevalence of cirrhosis. These comparisons should be interpreted descriptively, particularly given the variable availability of routinely collected laboratory measurements in the external cohort.

**Figure 1:**
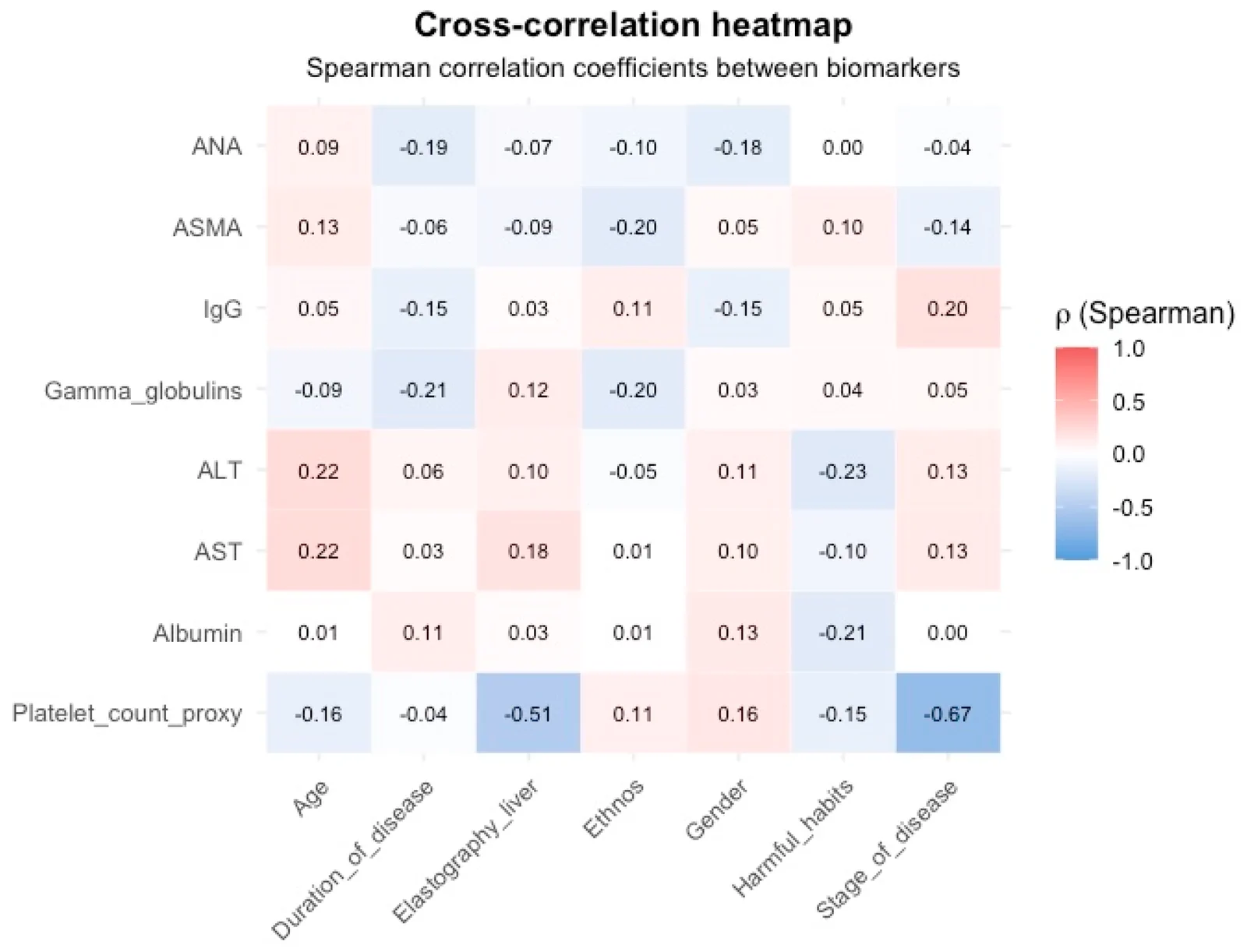
Correlation plot for all patients. This figure shows a correlation heat map illustrating the relationships between clinical, biochemical, and immunological parameters in patients with autoimmune hepatitis. The most pronounced negative correlation was found between platelet count and fibrosis stage (ρ = −0.67). In addition, platelet count was negatively correlated with liver stiffness as measured by transient elastography (ρ = −0.51).

In this study, a correlation matrix (Figure 1) was created to examine the relationships among biochemical and immunological parameters in patients with autoimmune hepatitis. Each cell of the matrix represents the strength and direction of association between two variables, allowing the identification of biomarker patterns associated with cirrhosis status. This approach provides an initial overview of how key indicators—including platelet count, elastography liver, ALT, IgG, and γ-globulins—are associated with each other and with cirrhosis status.

Liver stiffness showed a clear positive correlation with histological fibrosis, while γ-globulins and IgG demonstrated moderate positive correlations with both liver stiffness and disease stage, and autoantibodies (ANA and ASMA) showed only moderate associations with clinical and biochemical parameters.

**Figure 2:**
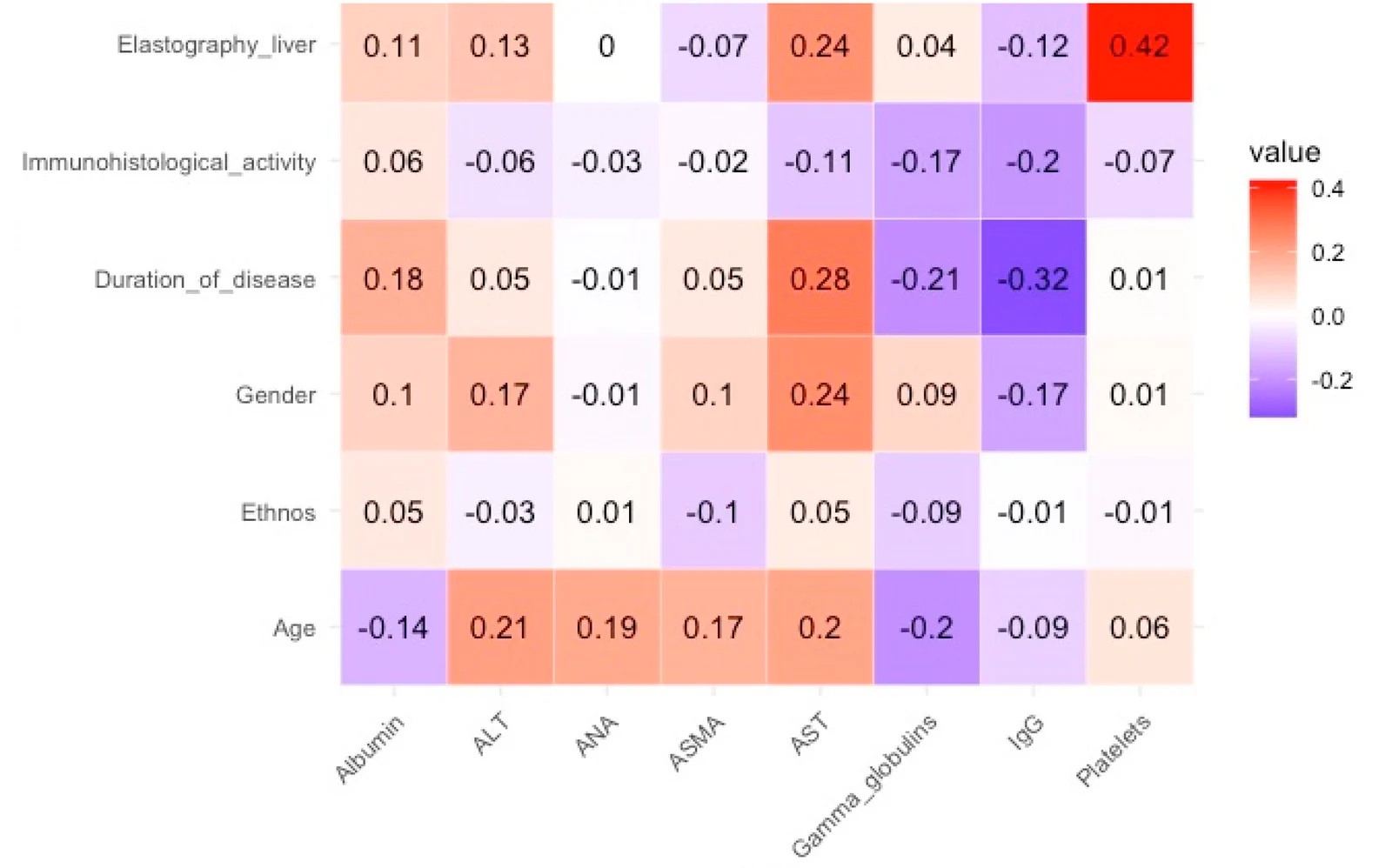
Correlation plot for patients without cirrhosis.

In patients without cirrhosis (Figure 2), the correlation matrix was characterized primarily by weak and moderate correlations among the variables under study. Platelet count showed a moderate positive correlation with liver stiffness (ρ = 0.42). ALT and AST showed weak positive correlations with disease duration (ρ ≈ 0.28) and age (ρ ≈ 0.21). IgG levels were negatively correlated with disease duration (ρ = −0.32), while γ-globulins showed a weak negative correlation with AST (ρ = −0.21). The autoantibodies ANA and ASMA were associated with clinical and laboratory parameters primarily through moderate correlations.

**Figure 3:**
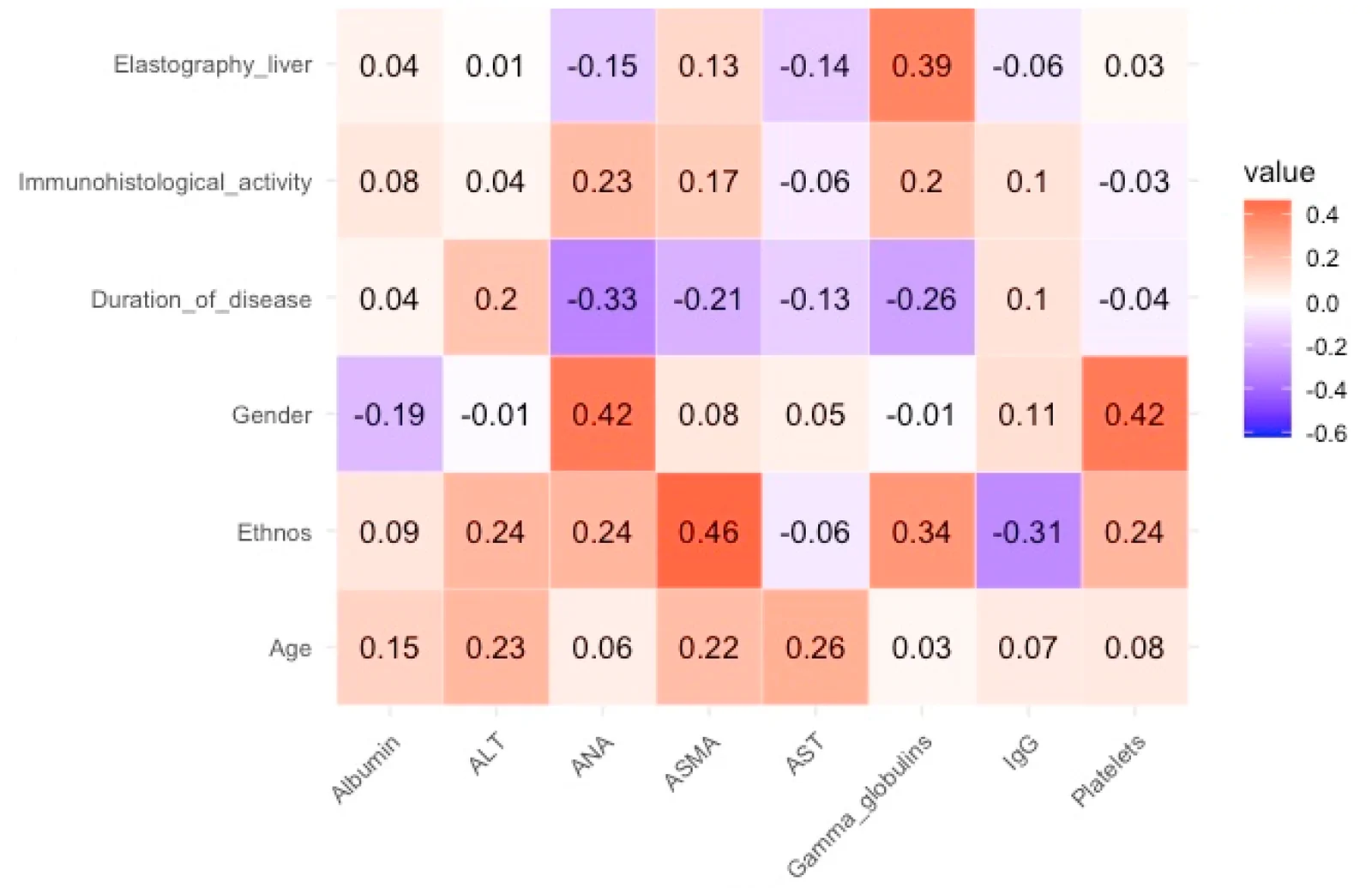
Correlation plot for patients with cirrhosis.

In the subgroup of patients with cirrhosis (Figure 3), the correlation matrix revealed a close relationship between biochemical and immunological markers. Compared with patients without cirrhosis, the correlations were stronger and often reversed. A notable finding was the strong negative correlation between harmful habits and AST activity (ρ = −0.62). Liver stiffness, as measured by elastography, positively correlated with γ-globulins (ρ = 0.39), while autoantibodies (ANA, ASMA) showed moderate associations with gender and ethnocultural background (ρ ≈ 0.42–0.46). Age showed a weak but positive correlation with ALT and AST (ρ ≈ 0.23–0.26), whereas disease duration correlated negatively with ANA levels (ρ = −0.33), and AST correlated negatively with γ-globulins (ρ = −0.16).

**Figure 4:**
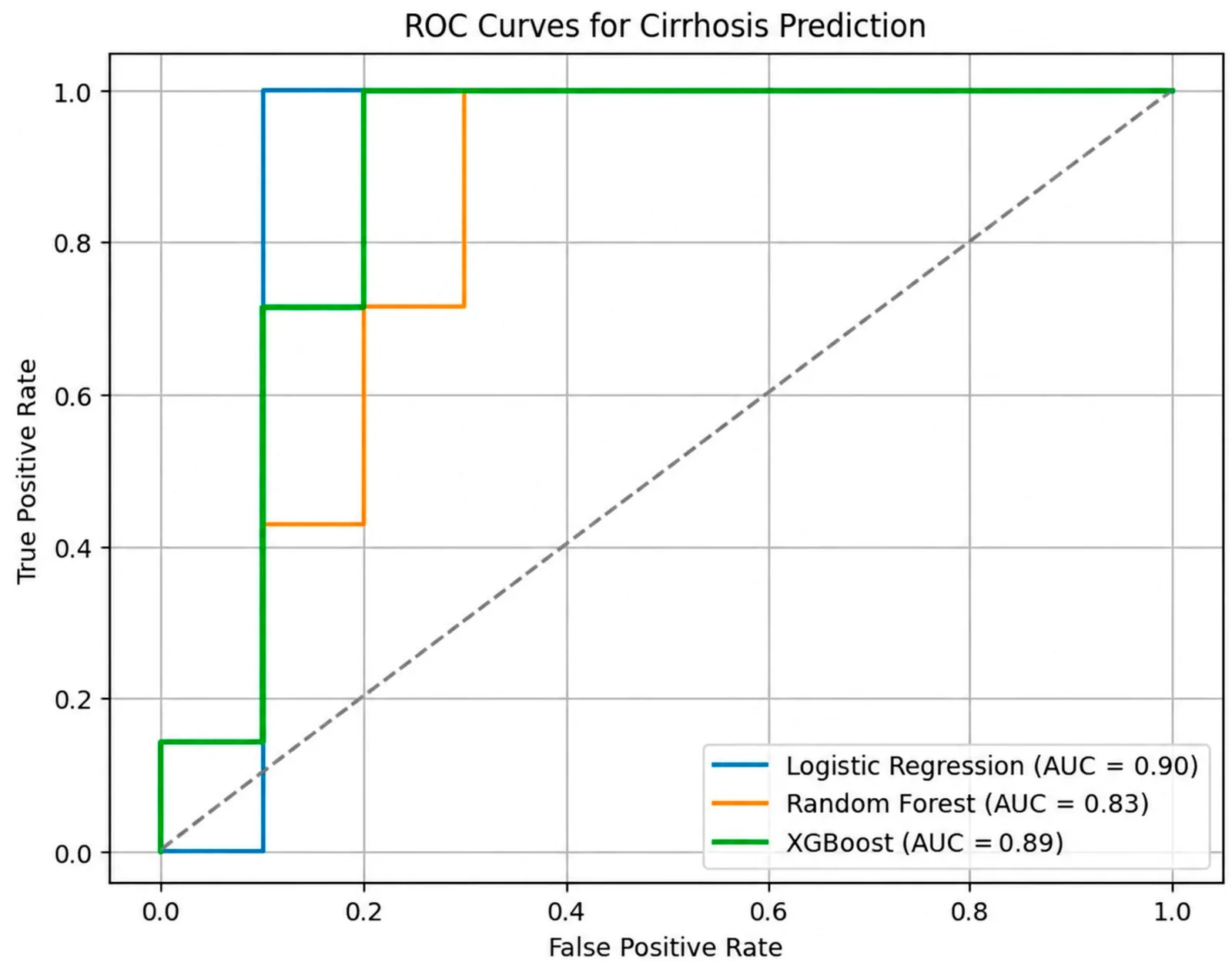
ROC curves. The gray dashed diagonal line represents chance-level discrimination (AUROC = 0.50).

Based on these patterns, we trained supervised machine learning models to classify cirrhosis status using integrated clinical, biochemical, immunological, and elastography data (Table 3). Logistic regression achieved an accuracy of 0.82, an F1 score of 0.77, and the highest AUC value (0.90), indicating high linear discriminatory power. The Random Forest model showed less favorable results: an accuracy of 0.71, an F1 score of 0.67, and an AUC of 0.83. XGBoost provided the best overall balance of performance, achieving an accuracy of 0.88, an F1 score of 0.88, and an AUC of 0.89 (Figure 4).

**Figure 5:**
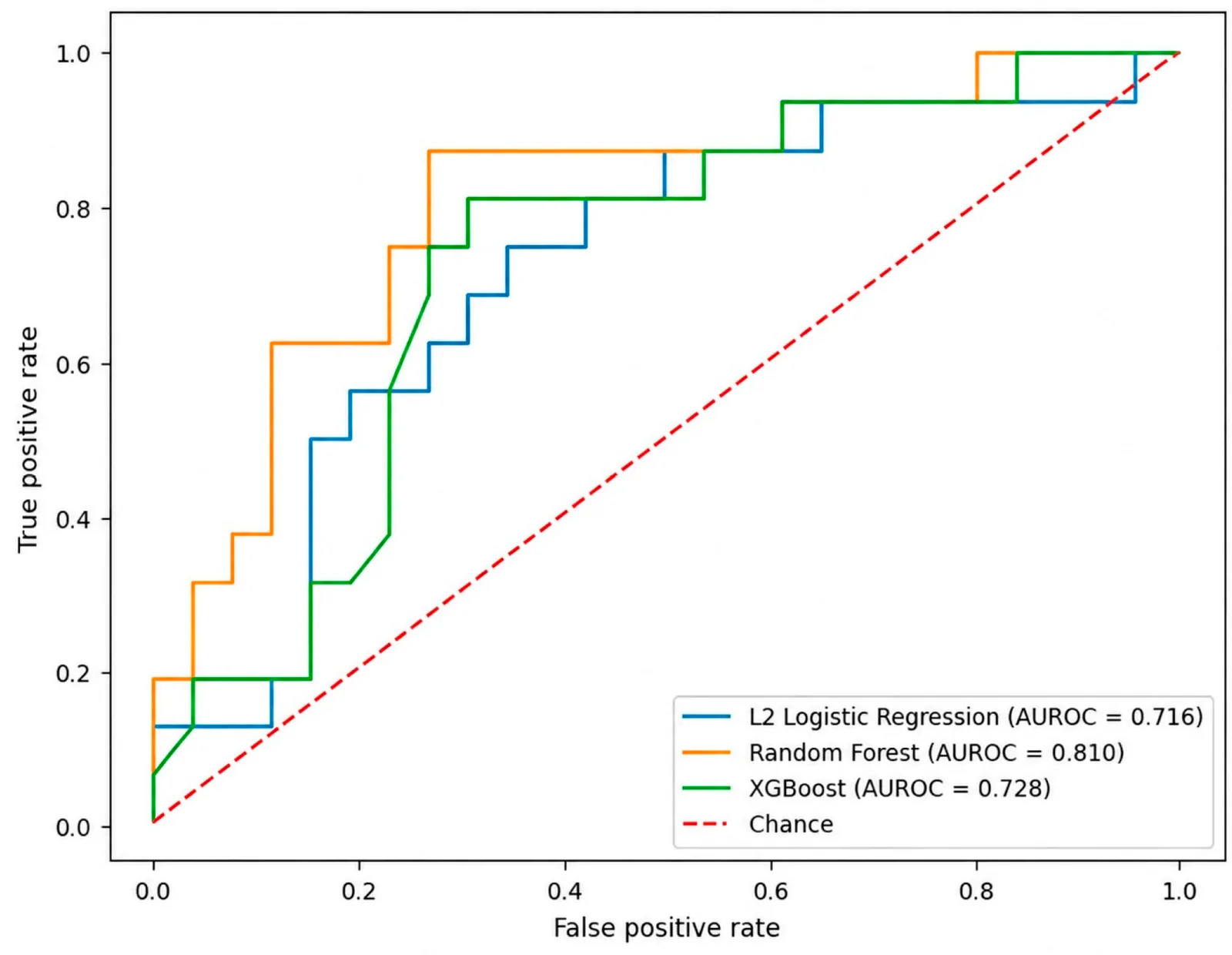
External ROC.

**Figure 6:**
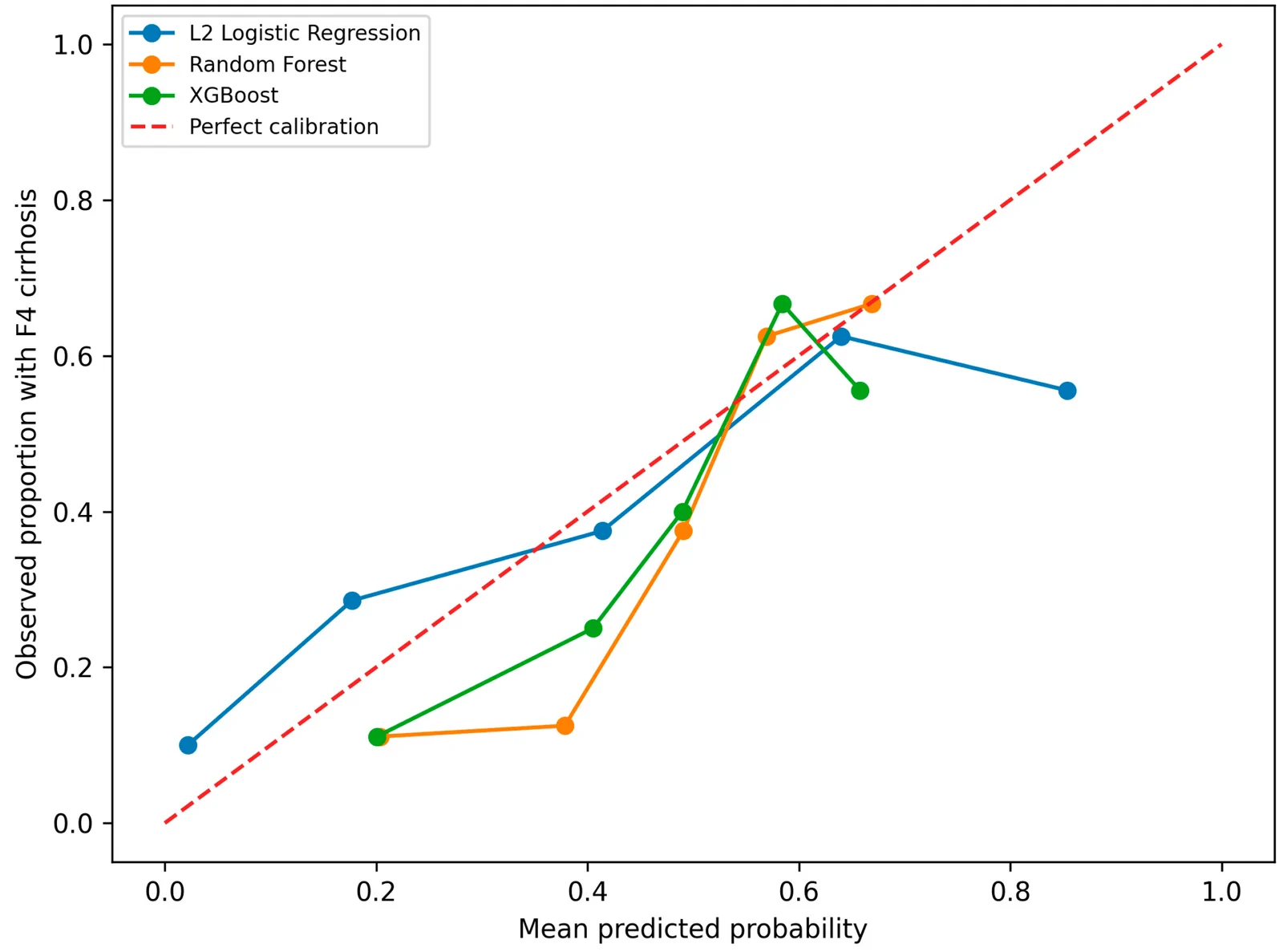
Calibration plot in the external validation cohort.

External validation ROC curves are presented in Figure 5, and calibration results are shown in Figure 6.

The ROC curves showed higher discrimination for Logistic Regression and XGBoost than for Random Forest in the original held-out test set; however, these estimates should be interpreted cautiously because the test set contained only 17 patients.

To assess the stability of the internal results beyond a single train-test split, an additional repeated stratified five-fold cross-validation with 20 repeats was performed. Mean AUROC was 0.878 ± 0.021 for logistic regression with L2 regularization, 0.914 ± 0.015 for Random Forest, and 0.896 ± 0.015 for XGBoost. The corresponding empirical 2.5th–97.5th percentile ranges were 0.842–0.913, 0.884–0.935, and 0.863–0.917, respectively. These results indicate that discrimination remained relatively stable across repeated data partitions, although the small development cohort remained an important source of uncertainty.

Table 4 summarizes the operating characteristics derived from the confusion matrix. Sensitivity reflects the proportion of true diseased cases correctly identified (TP/(TP+FN)), whereas specificity reflects the proportion of true non-diseased cases correctly recognized (TN/(TN+FP)). PPV indicates the probability that a positive prediction corresponds to true disease (TP/(TP+FP)), and NPV indicates the probability that a negative prediction corresponds to true absence of disease (TN/(TN+FN)). In our test set, XGBoost achieved a sensitivity of 1.000 (FN = 0), indicating no missed positive cases, with a specificity of 0.800 (FP = 2). Logistic regression showed higher specificity (0.900; FP = 1) but lower sensitivity (0.714; FN = 2). Because the test sample was small, 95% confidence intervals were wide and should be interpreted cautiously.

External validation performance is summarized in Table 5.

In the primary histology-matched external validation cohort (Table 5; Figure 5), Random Forest showed the highest numerical discrimination, with an AUROC of 0.810 (95% CI, 0.653–0.933), followed by XGBoost (AUROC 0.728, 95% CI, 0.566–0.878) and logistic regression with L2 regularization (AUROC 0.716, 95% CI, 0.545–0.875). Random Forest also had the lowest Brier score (0.188) and the highest sensitivity (0.875) at the fixed probability threshold of 0.5. Given the small external cohort and overlapping confidence intervals, numerical differences between models should not be interpreted as evidence of superiority.

Quantitative calibration analysis (Figure 6) showed an intercept of −0.288 (95% CI, −1.031 to 0.402) and slope of 0.272 (95% CI, 0.019–1.001) for logistic regression with L2 regularization; an intercept of −0.400 (95% CI, −1.511 to 0.252) and slope of 1.948 (95% CI, 0.776–5.627) for Random Forest; and an intercept of −0.364 (95% CI, −1.174 to 0.302) and slope of 1.256 (95% CI, 0.366–3.147) for XGBoost. The wide confidence intervals indicate substantial uncertainty in calibration estimates in this small validation cohort. The probability threshold of 0.5 was used as a fixed descriptive operating point and was not optimized for clinical decision-making.

For comparison with clinically established non-invasive measures, elastography stage alone achieved an AUROC of 0.745 (95% CI, 0.602–0.883), APRI an AUROC of 0.691 (95% CI, 0.528–0.845), and FIB-4 an AUROC of 0.715 (95% CI, 0.561–0.853). Although the full Random Forest model had a numerically higher AUROC than elastography alone (0.810 vs. 0.745), the difference was not statistically significant (ΔAUROC 0.065, 95% CI, −0.061 to 0.184; *p* = 0.335).

In an exploratory ablation analysis, removal of ANA, ASMA, IgG, and γ-globulins reduced Random Forest AUROC from 0.810 to 0.748 (ΔAUROC 0.063, 95% CI, 0.005–0.122; nominal *p* = 0.037). Given the small sample size, multiple exploratory comparisons, and substantial missingness of immunological measurements, this result should be regarded as hypothesis-generating rather than confirmatory.

**Figure 7:**
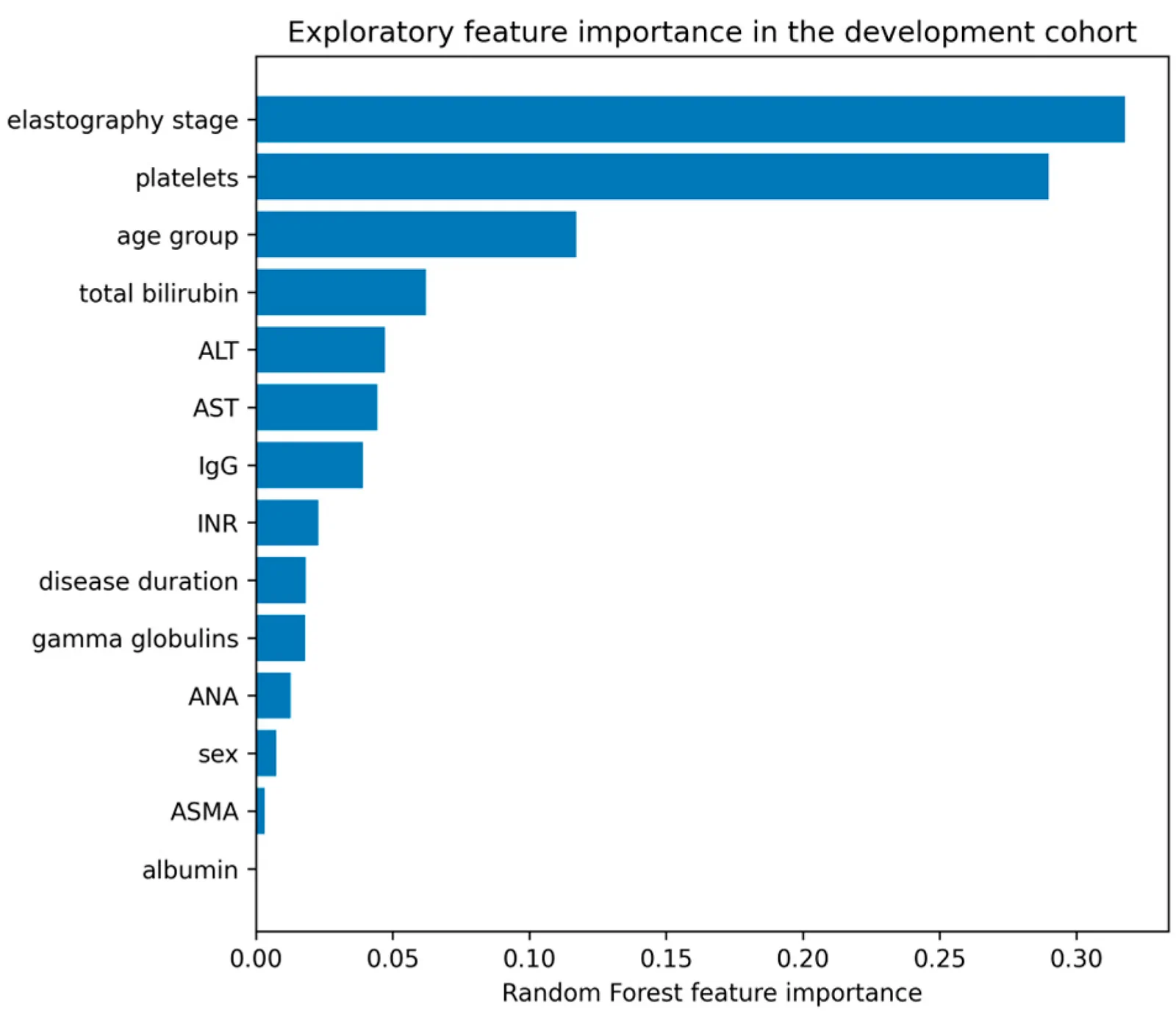
Random Forest feature importance.

Feature-importance and SHAP analyses were regenerated using the final Random Forest model and the harmonized predictor set. The analyses were used to characterize the relative contribution of the recorded variables to model output (see Figure 7).

Elastography stage and platelet count were among the most influential model-associated features, followed by age group, total bilirubin, and other laboratory and immunological variables. Feature importance reflects the contribution of variables to the fitted model and should not be interpreted as evidence of biological causality.

**Figure 8:**
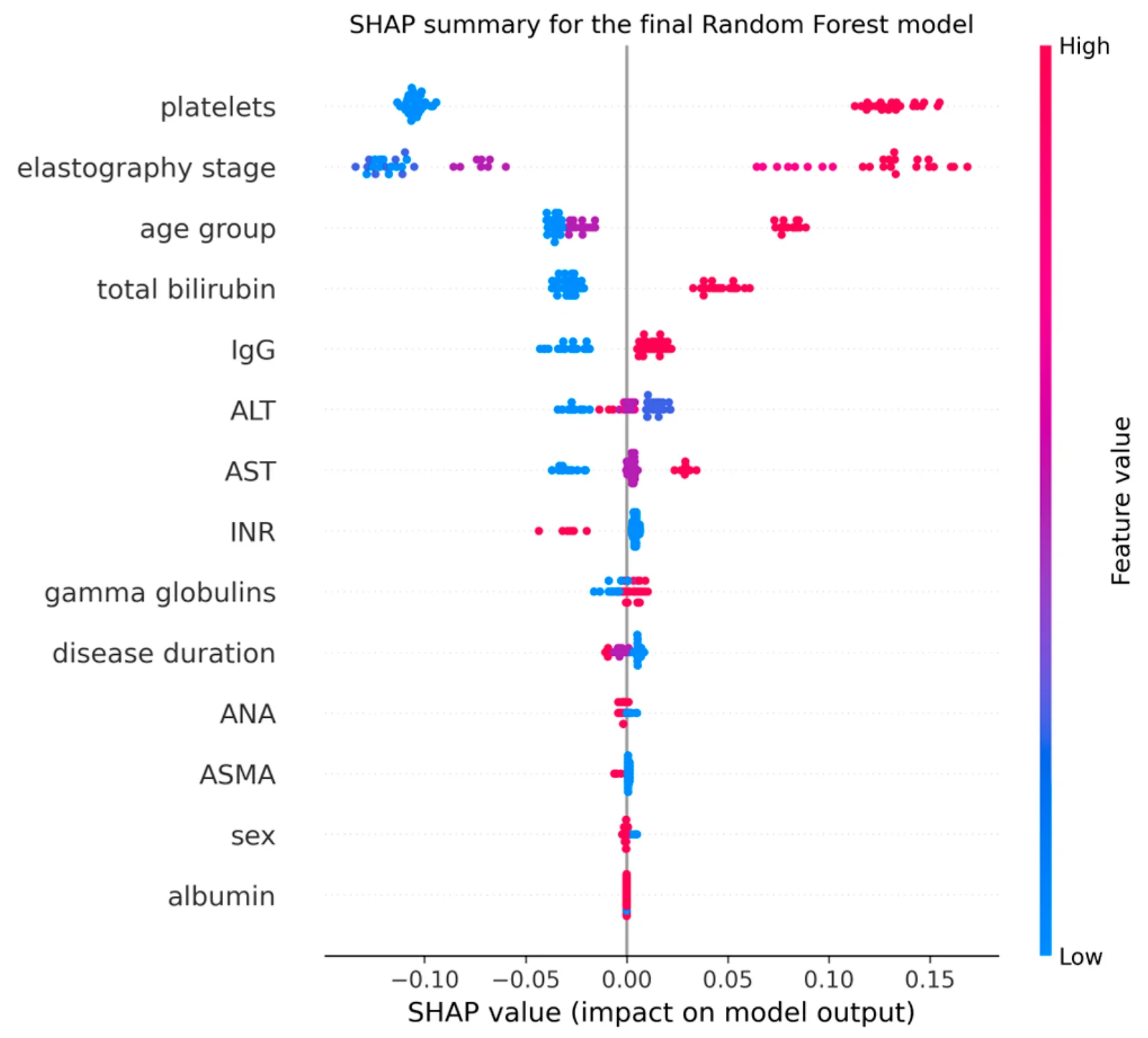
SHAP summary plot.

The SHAP analysis (Figure 8) provided a complementary view of how individual variables contributed to Random Forest predictions. The direction and magnitude of SHAP values indicate how recorded feature values shifted the model output toward classification as cirrhosis or non-cirrhosis. Because several predictors were represented using categorical or encoded values, the SHAP patterns should be interpreted as model behavior rather than direct biological effects.

## 4. Discussion

It is known that chronic liver damage triggers the activation of liver cells and inflammatory cells, leading to inflammation and oxidative stress due to the production of mediators. This contributes to increased collagen production and the accumulation of extracellular matrix, causing liver fibrosis and cirrhosis [1].

Since these processes are multifactorial in nature, no single biomarker is capable of fully reflecting disease progression. Our study showed that a combination of indicators reflecting different aspects of AIH was informative for the classification of cirrhosis status. The most influential model-associated features included liver stiffness, platelet count, and immunological markers, consistent with the multifactorial nature of AIH and the potential value of integrating routine clinical and laboratory information.

According to our findings, liver stiffness, as measured by transient elastography, showed the strongest contribution to the model-based classification of cirrhosis status, consistent with its established role as a non-invasive marker of liver fibrosis in AIH.

Similar studies have shown that transient elastography is one of the most accurate noninvasive methods for assessing the stage of fibrosis in autoimmune hepatitis, although it cannot completely replace a liver biopsy in evaluating inflammatory activity and remission [17].

Platelet count also emerged as one of the influential model-associated features for cirrhosis in our model. This is consistent with data on the pathophysiology of chronic liver diseases, according to which thrombocytopenia is considered one of the most sensitive markers of progressive fibrosis and cirrhosis. The development of thrombocytopenia is multifactorial in nature and is associated with portal hypertension, hypersplenism, and reduced thrombopoietin synthesis in the liver [18]. Taken together, these mechanisms are consistent with the observed contribution of platelet count to cirrhosis classification in our model.

In addition to structural changes in the liver and platelet count, immunological and biochemical parameters are of significant importance in AIH. In this study, correlation analysis revealed moderate positive correlations between γ-globulins and IgG, a weak correlation between aminotransferases and liver stiffness as well as disease stage, and moderate associations between autoantibodies and clinical and biochemical parameters.

It is known that hypergammaglobulinaemia with elevated IgG levels may reflect persistent immuno-inflammatory activity in AIH [19]. However, elevated IgG levels are not specific to AIH and are observed in other liver diseases, which necessitates interpretation in conjunction with clinical, biochemical, and diagnostic data [20]. In our study, the inclusion of IgG and γ-globulins in the classification models suggests that immunological markers may provide complementary information for the classification of cirrhosis status in patients with AIH.

Despite the substantial contribution of individual indicators to the models, none of them alone is sufficient to fully characterize cirrhosis status in patients with AIH. Liver stiffness reflects the accumulation of fibrous tissue, whilst platelet count reflects the progression of portal hypertension; in contrast, immunological parameters—in particular γ-globulins and IgG—characterise the disease’s ongoing immunological activity. In contrast to patients with cirrhosis, those without cirrhosis showed a weak or moderate correlation between laboratory parameters. Despite the contribution of individual variables to the models, no single marker fully characterized cirrhosis status in patients with AIH. Liver stiffness, platelet count, biochemical variables, and immunological markers represent complementary aspects of the clinical phenotype. The subgroup correlation analyses showed predominantly weak-to-moderate associations among these variables. These correlations should be interpreted as descriptive associations and not as evidence of causal relationships or disease mechanisms.

This is precisely why the use of machine learning methods is particularly promising in the case of autoimmune hepatitis, as they take into account the relationships between numerous clinical, biochemical, immunological and diagnostic parameters, which is important given the heterogeneous nature of autoimmune hepatitis.

Only a few studies have applied machine learning specifically to autoimmune hepatitis, with most existing models focusing on viral or metabolic liver diseases. The three machine-learning approaches showed different performance patterns across internal and external evaluation. Although Logistic Regression with L2 regularization and XGBoost performed well in the original held-out test set, the additional repeated cross-validation analysis demonstrated relatively stable discrimination across alternative data partitions. In the primary histology-matched external validation cohort, Random Forest showed the highest numerical discrimination (AUROC 0.810), whereas Logistic Regression with L2 regularization and XGBoost achieved AUROCs of 0.716 and 0.728, respectively. However, the wide and overlapping confidence intervals preclude conclusions regarding the superiority of one model over another.

Despite the limited number of studies focusing on AIH, the effectiveness of ML approaches has been confirmed in another cohort of patients with chronic hepatitis B, where fibrosis markers, including liver stiffness and platelet count, were also leading predictors of adverse outcomes [21].

Furthermore, in a recent study [22], artificial neural network models were developed and validated to predict short-term mortality (28-day and 90-day) in hospitalised patients with acute decompensated liver cirrhosis. Compared with the traditional MELD-Na and MELD 3.0 scales, the machine learning models demonstrated significantly higher accuracy (AUC 0.81 and 0.84). These results indicate that machine learning tools can provide a more reliable prediction of short-term mortality in cirrhosis than standard assessment systems.

External validation provides an important assessment of model transportability, particularly given the small development cohort. Performance was lower and more uncertain outside the original development sample. In the primary independent histology-matched validation cohort, Random Forest showed the highest numerical AUROC of 0.810 (95% CI, 0.653–0.933), compared with 0.728 (95% CI, 0.566–0.878) for XGBoost and 0.716 (95% CI, 0.545–0.875) for Logistic Regression with L2 regularization. Random Forest also showed the lowest Brier score (0.188). Nevertheless, confidence intervals were wide and overlapping, indicating substantial uncertainty and limited precision of the external estimates. The reduction and variability in performance relative to the internal analyses emphasize the remaining risk of optimism and the need for larger independent validation cohorts. This decline is attributable to clinical and methodological factors, as external validation reflects differences in patient characteristics, clinical practice, laboratory measurements, data completeness and the distribution of diseases across centers.

Comparison with established non-invasive measures further contextualized the model performance. Elastography stage alone achieved an AUROC of 0.745, while APRI and FIB-4 achieved AUROCs of 0.691 and 0.715, respectively. Although the full Random Forest model showed a numerically higher AUROC than elastography alone (0.810 vs. 0.745), the incremental difference was not statistically clear (ΔAUROC 0.065, 95% CI, −0.061 to 0.184; *p* = 0.335). Therefore, the present results do not establish that the multivariable machine-learning model provides superior discrimination to elastography alone.

The exploratory ablation analysis suggested a possible contribution of immunological variables: removal of ANA, ASMA, IgG, and γ-globulins reduced Random Forest AUROC from 0.810 to 0.748. However, given the small validation sample, substantial missingness of immunological measurements, multiple exploratory comparisons, and the absence of multiplicity adjustment, this finding should be considered hypothesis-generating rather than confirmatory.

Calibration assessment also demonstrated uncertainty in the transportability of predicted probabilities. Although Random Forest had the lowest Brier score, calibration intercepts and slopes had wide confidence intervals across all three models. These findings indicate that discrimination alone does not establish reliable probability estimation and that calibration should be reassessed in larger prospective cohorts before clinical use. The fixed probability threshold of 0.5 used in this study was retained only as a descriptive operating point and should not be interpreted as an optimized clinical decision threshold.

The present study extends our previous work [13] by specifically focusing on histological cirrhosis (F4 versus F0–F3), incorporating immunological and elastography-related variables, and adding independent external validation. In contrast to the previous study, which evaluated advanced fibrosis (F3–F4 versus F0–F2) and was limited to internal validation, the present analysis addresses a distinct classification target and includes primary histology-matched external validation.

This study has several limitations. Firstly, the development cohort included only 55 patients with biopsy-confirmed AIH. Given the number of candidate features relative to the sample size, overfitting cannot be excluded and may have resulted in overestimation of model performance in the development cohort. Secondly, although an independent external validation cohort was used, the study remained retrospective, and differences in clinical assessment, laboratory measurements, data completeness, and the definition of cirrhosis between centres may have influenced the external efficacy. Thirdly, information on cirrhosis status was missing for a subgroup of patients in the external database; therefore, only patients with available information on disease outcome were included in the external validation analysis.

Fourthly, the calibration was suboptimal and requires further evaluation in larger prospective cohorts. Fifthly, antibodies against SLA and additional imaging techniques or molecular biomarkers are not available in routine practice, although they may provide additional discriminatory value in future models. Finally, the presented models should not yet be considered ready for direct clinical implementation. Prospective multicentre validation and an assessment of clinical utility are required before integration into routine hepatological practice.

Despite these limitations, our findings support further evaluation of the machine-learning models integrating routine laboratory, immunological and elastography markers for the cirrhosis classification in AIH.

## 5. Conclusions

This study developed and externally evaluated interpretable machine-learning models for the classification of prevalent cirrhosis in patients with autoimmune hepatitis using routinely available clinical, biochemical, immunological, and elastography-related variables. In the original internal held-out analysis, Logistic Regression with L2 regularization and XGBoost showed good discrimination, while additional repeated cross-validation demonstrated relatively stable internal performance across alternative data partitions.

In the primary independent histology-matched external validation cohort, Random Forest showed the highest numerical discrimination, with an AUROC of 0.810, followed by XGBoost and Logistic Regression with L2 regularization. However, the wide and overlapping confidence intervals indicate substantial uncertainty, and the observed differences between models should not be interpreted as evidence of superiority. Furthermore, although the full Random Forest model numerically outperformed elastography alone, a statistically clear incremental advantage was not demonstrated.

Feature-importance and SHAP analyses identified liver stiffness, platelet count, and several clinical, biochemical, and immunological variables as influential model-associated features. These findings describe model behavior and statistical associations and should not be interpreted as evidence of causal biological effects. Overall, this study provides preliminary evidence of model transportability in an independent clinical setting. Larger prospective multicenter studies are required to confirm discrimination, calibration, incremental predictive value, and clinical utility before routine implementation.

## Figures and Tables

**Table 1:** Immunological and biochemical indicators in autoimmune hepatitis.

Parameter	Description
ANA	A class of antibodies capable of binding to nuclear components of cells—proteins, DNA, RNA, and nucleoprotein complexes—is a diagnostic marker of systemic autoimmune diseases.
ASMA	Autoantibodies directed against microfilaments, microtubules, and intermediate filaments of human smooth muscle.
IgG	Glycoprotein necessary for protecting the body from pathogen invasion.
Gamma globulins	A fraction of serum globulin that neutralizes pathogens by recognizing antigens.
ALT	A liver enzyme involved in amino acid metabolism.
AST	A liver enzyme involved in amino acid metabolism.
Total bilirubin	Marker of hepatic excretory dysfunction and cholestasis.
Platelets	Blood cell elements involved in clot formation.
Albumin	A liver-synthesized protein that reflects the severity of hepatic synthetic dysfunction.

**Table 2:** Comparison of baseline characteristics between the development and external validation cohorts.

Characteristic	Development Cohort (*n* = 55)	External Validation Cohort (*n* = 154)	*p* Value
Demographic characteristics			
Age ≤ 44 years, *n* (%)	23 (41.8)	50 (32.5)	
Age 45–59 years, *n* (%)	18 (32.7)	53 (34.4)	0.561 *
Age 60–74 years, *n* (%)	14 (25.5)	50 (32.5)	
Age 75–90 years, *n* (%)	0 (0.0)	1 (0.6)	
Female sex, *n* (%)	47 (85.5)	133 (86.4)	0.867
Asian ethnicity, *n* (%)	46 (83.6)	133 (86.4)	0.620
Immunological characteristics			
ANA-positive, *n* (%)	44 (80.0)	133 (86.4)	0.261
Elevated IgG, *n*/*N* (%)	35/55 (63.6)	59/103 (57.3)	0.438
Elevated γ-globulins, *n*/*N* (%)	24/55 (43.6)	50/63 (79.4)	<0.001
Biochemical and hematological characteristics			
ALT above reference range, *n*/*N* (%)	39/51 (76.5)	127/149 (85.2)	0.150
AST above reference range, *n*/*N* (%)	40/52 (76.9)	117/140 (83.6)	0.289
Elevated total bilirubin, *n*/*N* (%)	20/50 (40.0)	71/146 (48.6)	0.291
Low albumin, *n*/*N* (%)	2/29 (6.9)	21/88 (23.9)	0.046
Low platelet count, *n*/*N* (%)	24/55 (43.6)	67/144 (46.5)	0.714
Liver disease characteristics			
Cirrhosis, *n* (%)	23 (41.8)	94 (61.0)	0.014

Note: Data are presented as *n* (%) or *n*/*N* (%) as appropriate, where *N* represents the number of patients with available data for the respective variable. In the external validation cohort, IgG and γ-globulin measurements were available for 103/154 (66.9%) and 63/154 (40.9%) patients, respectively. Missing measurements were treated as missing data and were not considered normal or negative findings. *p* values represent descriptive between-cohort comparisons. * *p* value for age represents the overall comparison across age categories.

**Table 3:** Performance of machine learning models for cirrhosis classification.

Model	Accuracy	F1-Score	AUC
Logistic Regression	0.82	0.77	0.90
Random Forest	0.71	0.67	0.83
XGBoost	0.88	0.88	0.89

**Table 4:** Operating characteristics on the held-out test set (threshold = 0.5). The 95% confidence intervals (95% CI) were calculated using the Wilson score method.

Model	Sensitivity (95% CI)	Specificity (95% CI)	PPV (95% CI)	NPV (95% CI)	TP	FP	TN	FN
Logistic Regression	0.714 [0.359, 0.918]	0.900 [0.596, 0.982]	0.833 [0.436, 0.970]	0.818 [0.523, 0.949]	5	1	9	2
Random Forest	0.714 [0.359, 0.918]	0.700 [0.397, 0.892]	0.625 [0.306, 0.863]	0.778 [0.453, 0.937]	5	3	7	2
XGBoost	1.000 [0.646, 1.000]	0.800 [0.490, 0.943]	0.778 [0.453, 0.937]	1.000 [0.676, 1.000]	7	2	8	0

**Table 5:** External validation performance of machine-learning models in an independent AIH cohort.

Model	AUROC (95% CI)	PR-AUC (95% CI)	Brier (95% CI)	Sensitivity (95% CI)	Specificity (95% CI)	PPV (95% CI)	NPV (95% CI)
Logistic Regression with L2 regularization	0.716 (0.545–0.875)	0.612 (0.398–0.838)	0.225 (0.140–0.316)	0.625 (0.386–0.815)	0.692 (0.500–0.835)	0.556 (0.337–0.754)	0.750 (0.551–0.880)
Random Forest	0.810 (0.653–0.933)	0.744 (0.520–0.917)	0.188 (0.151–0.227)	0.875 (0.640–0.965)	0.692 (0.500–0.835)	0.636 (0.430–0.803)	0.900 (0.699–0.972)
XGBoost	0.728 (0.566–0.878)	0.607 (0.378–0.836)	0.210 (0.170–0.252)	0.688 (0.444–0.858)	0.731 (0.539–0.863)	0.611 (0.386–0.797)	0.792 (0.595–0.908)

## Data Availability

The data presented in this study are not publicly available due to ongoing research and ethical restrictions to protect patient confidentiality. De-identified data may be available from the corresponding author on reasonable request and with appropriate institutional approvals.
